# Intra-thyroid thyroglossal duct cyst: a case report and review of the literature

**DOI:** 10.1093/jscr/rjaf152

**Published:** 2025-03-25

**Authors:** Sarra Ben Rejeb, Yasmine Chaabane, Ameni Hachicha, Sinda Turki

**Affiliations:** Pathology Department, Security Forces Hospital, Tahar Ben Achour Street, 1050, Marsa, Tunisia; Pathology Department, Security Forces Hospital, Tahar Ben Achour Street, 1050, Marsa, Tunisia; Ear, Nose and Throat Department, Security Forces Hospital, Tahar Ben Achour Street, 1050, Marsa, Tunisia; Ear, Nose and Throat Department, Security Forces Hospital, Tahar Ben Achour Street, 1050, Marsa, Tunisia

**Keywords:** thyroid surgery, pathology, thyroglossal duct cyst

## Abstract

Thyroglossal duct cyst (TGDC) is the most common congenital anomaly of thyroid gland. TGDC are ectopic remnants mainly occurring in the midline of the neck, rarely at other sites. Intrathyroidal thyroglossal duct cysts (ITTGDC) are extremely rare and may be confused with thyroid cancer. We herein described a rare case of ITTGDC with a review of the literature. A 47-year-old woman presented with a painless, mobile midline neck mass of 2 months duration. Clinical and imaging investigations suspected thyroid nodule that was classified EU-TIRADS3 on ultrasound. The cytology from fine needle aspiration was categorized as benign. A lobectomy was performed. The histopathologic examination showed a macrovesicular adenoma. Unexpectedly, a 1.2 cm ductal cystic structure with pseudostratified ciliated columnar epithelium was identified adjacent to the thyroid pseudocapsule. It was diagnosed as ITTDGC. Mild lymphocytic thyroiditis was also present. This case highlights the diagnostic challenge of ITTGDC, particularly given its rarity with only few reports documented in the literature. This report, accompanied by a review of the literature, adds to the limited documentation of ITTGDC and underscores the need for awareness of this rare entity among clinicians and pathologists.

## Introduction

Thyroglossal duct cyst (TGDC) is the most common congenital anomaly of the thyroid gland1. It is related to the persistence of an embryological structure that normally regresses after thyroid descent. These remnants are typically located in the midline of the neck leading to abnormal congenital neck mass [1]. Although the midline of the neck is the typical site, TGDC has been described in uncommon locations, and of these, intra-thyroid TGDC (ITTGDC) is extremely rare with only few cases documented in the literature [2–5]. The clinical presentation of ITTGDC can mimic other thyroid pathologies, including nodules or malignancies, making accurate diagnosis challenging [6]. We herein described a case of ITTGDC incidentally discovered on pathological examination and provided a comprehensive review of the literature.

## Case report

**Figure 1:**
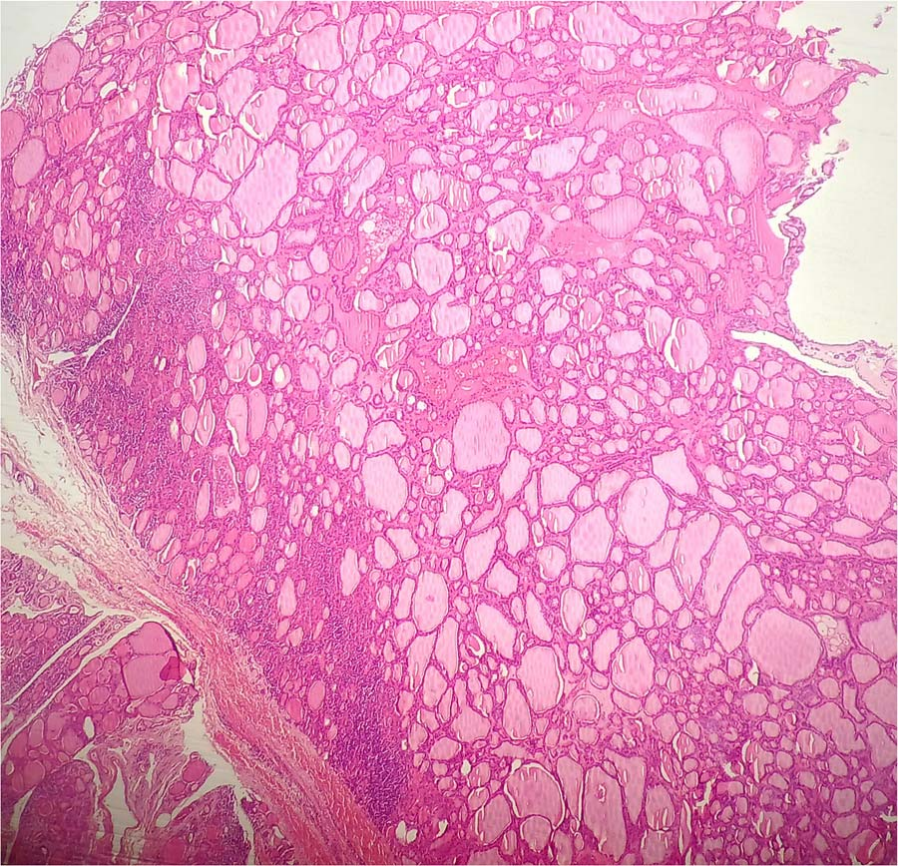
HE × 4: Thyroid follicular adenoma.

**Figure 2:**
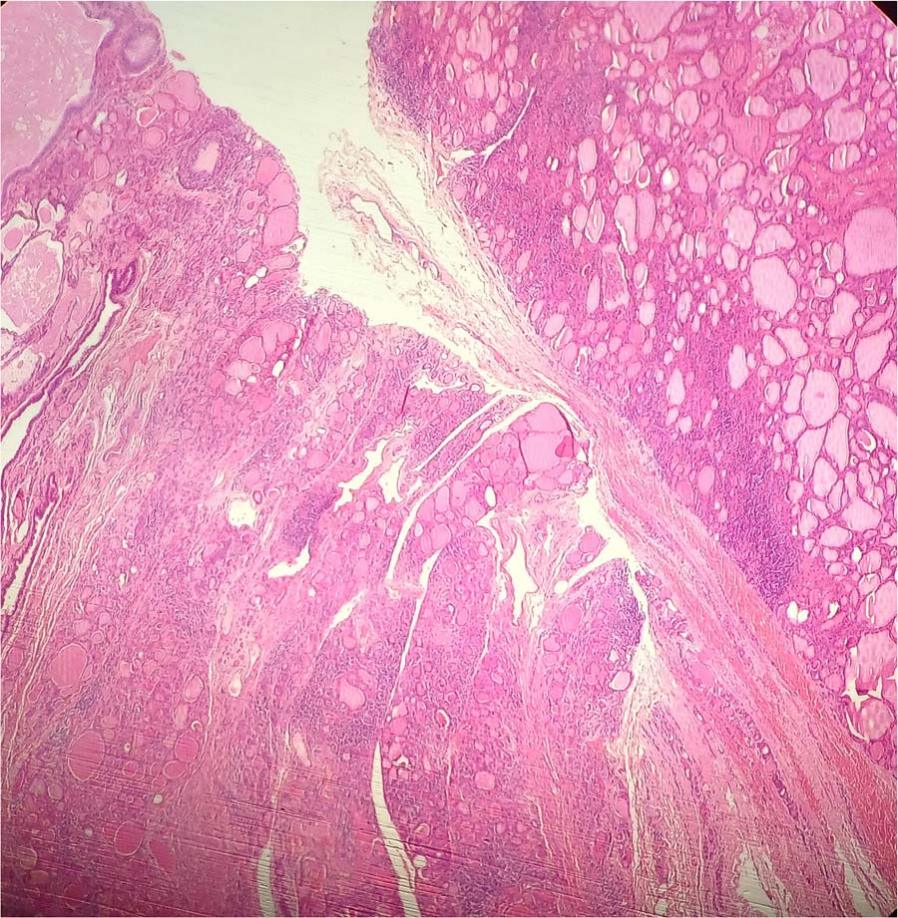
HE × 4 incidental finding of cystic duct structure in the adjacent thyroid gland tissue.

**Figure 3:**
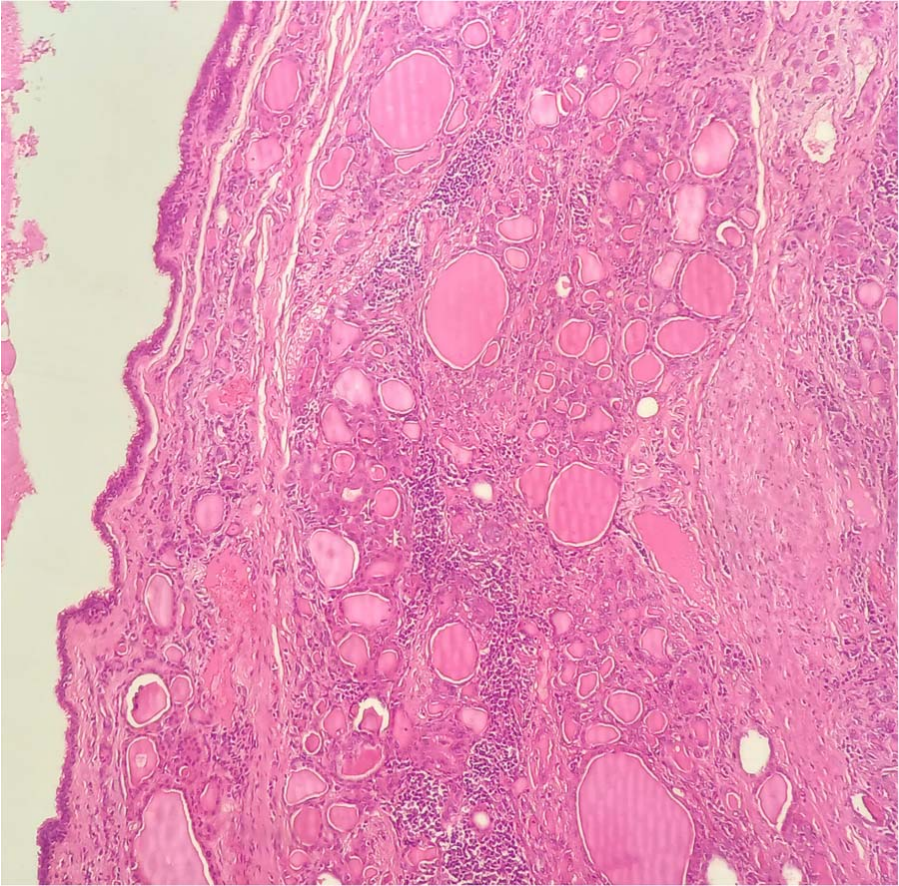
HE × 20: The cyst was lining with pseudostratified ciliated columnar cells and circumscribed with thyroid follicle within the cystic wall.

**Figure 4:**
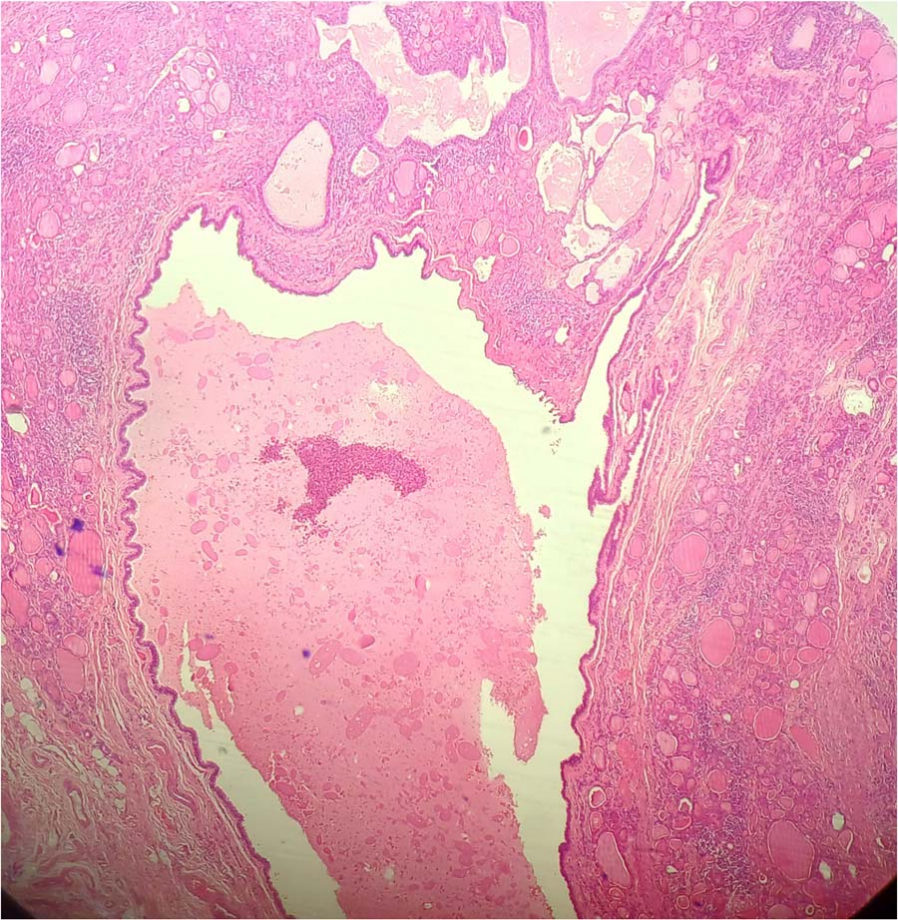
HE × 10: Intra-thyroid ductal cyst with eosinophilic material.

A 47-year-old woman with unremarkable medical history, presented with a 2 months history of neck mass along the midline. Physical examination revealed a 6 cm mobile, thyroid nodule without compressive symptoms. There was no associated erythema, fluctuance, tenderness or adhesion to adjacent structures. There was also no associated cervical lymphadenopathy. The rest of the physical exam was unremarkable. Laboratory tests including TSH, FT3, and FT4 revealed normal levels. On cervical ultrasound, there were a 62 mm left toto-lobar nodule classified as EU-TIRADS3, and an 11 mm right nodule, also classified as EU-TIRADS3. Fine-needle aspiration cytology concluded to a benign lesion classified as BETHESDA II. The patient reported respiratory discomfort in the supine position, leading to the indication for a lobectomy. Gross examination of the specimen revealed a toto-lobular nodule with a colloid appearance. It was well-circumscribed by a thin and regular fibrous capsule. The nodule measured 4 × 6 cm and exhibited areas of fibrous, whitish remodeling, as well as hemorrhagic and cystic changes. On microscopic examination, the nodule corresponded to a macro vesicular adenoma altered by fibrosis and hemorrhage. It was surrounded by a thin, regular, and intact fibrous capsule. However, at the periphery of the nodule, adjacent to the thyroid pseudo capsule, a 1.2 cm cystic ductal structure was observed. It showed pseudostratified ciliated columnar and squamous epithelial lining associated with thyroid follicles in the surrounding stroma (Figs 1 and 2). These thyroid follicles were bland with no papillary nuclear atypia or invasion (Fig. 3). The cyst was filled with a thin eosinophilic material (Fig. 4) Lymphocytic thyroiditis of mild severity was identified in the rest of the thyroid tissue. Based on these findings, the diagnosis of macro vesicular thyroid adenoma with ITTGDC and lymphocytic thyroiditis was made. After the surgery, the patient developed temporary dysphonia.

## Discussion

The thyroid gland originates early during fetal development, around the 4th week of gestation [1]. It begins as an endodermal thickening at the base of the tongue, also known as the foramen cecum. This primordial thyroid gland then descends caudally through the neck, following a midline path, to reach its final position in the anterior part of the neck, just below the larynx [1]. Throughout its migration, the thyroid remains temporarily connected to the tongue by a narrow canal called the thyroglossal duct [1]. By the 7th to 10th week of gestation, the thyroid gland completes its migration, and the thyroglossal duct normally involutes and disappears. However, in some cases, portions of the thyroglossal duct may persist, which are thought to be the source of TGDC. Although, TGDC typically occur along the midline of the neck, it can theoretically arise anywhere along the migratory path of the developing thyroid gland, from the base of the tongue to the mediastinum [1, 7, 8]. Even though extremely rare, TGDC may arise within the substance of the thyroid gland itself as evidenced by the reported cases in this study [1–4, 6, 9–11]. The exact incidence of ITTDC is still unknown. However based on the largest documented TGDC case series, Thompson *et al.* reported that 11 out of 685 cases (1.6%) were located within the thyroid gland [1]. In this literature review, by searching the terms “intra-thyroid”, “duct”, and “cyst” in the PubMed, Science Direct, and Elicit search engines, a total of 22 publications of case reports or series of histologically confirmed ITDTGC were identified (Table 1). Based on the cases reviewed in the literature, including 22 cases summarized in this study, several key clinical, cytological, and histological features are discussed. Clinically, ITTDCs appear to have a wide age range varying from 2 to 78 years-old [12]. The mean age of presentation was ⁓32 years, indicating that these cysts can manifest both in pediatric and adult populations. There was a slight male predominance, with 13 of the 23 cases (57%) occurring in men, which is inconsistent with the general trend of thyroid pathologies being more common in females. The present case was a female patient in her nearly fifties. Most patients presented with a solitary, palpable neck mass or thyroid nodule. Due to the intra-thyroid location, these cysts were frequently mistaken for benign thyroid nodules or cysts on initial evaluation. However, in three cases, the cyst was incidentally discovered during the systematic ultrasound or microscopic examination of thyroid specimens performed for other unrelated conditions highlighting the fact that ITTDCs may go unnoticed until they become large or symptomatic. Likewise, in this reported case, the cyst had no clinical manifestation and was incidentally discovered on microscopic examination of a lobo-isthmectomy specimen for thyroid adenoma. This finding highlights the importance of a thorough macroscopic examination and proper sampling of the thyroid gland specimens. On imaging, 70% of the reported cases showed a cystic nodule on ultrasound which was described as complex in two cases and calcified in another case [4, 6, 9–15]. In two cases, the lesion was described as an hypoechoic thyroid nodule [3]. In our case, the cyst was not detected on ultrasound which may be explained by its small size and its proximity to the large thyroid nodule detected on US. Pre-operative fine-needle aspiration (FNA) findings were available in 65% of the described cases. However, the results were often non-specific. Fine needle aspiration-biopsy (FNAB) typically yielded benign-appearing squamous cells or respiratory-type epithelial cells, with no indication of malignancy. In some cases, the FNAB findings led to a presumptive diagnosis of a benign thyroid cyst or colloid nodule, which delayed the recognition of the underlying ITTDC. This underscores the diagnostic challenges posed by FNA cytology in distinguishing ITTDC from other cystic thyroid lesions [2–4, 9, 11–14]. Notably, in one case, the cytological analysis suggested a branchial cleft cyst, further illustrating the overlap in cytological appearance between different types of congenital cysts and the necessity of histological confirmation [6]. In the present case, cytological findings were non-specific and were reported as BETHESDA II. Surgery was the definitive treatment in all reported cases with most patients undergoing thyroid lobectomy or hemithyroidectomy (16 cases), total thyroidectomy (one case). Simple surgical cyst excision was performed in 6 cases. In a minority of cases, where an external tract or extension toward the hyoid bone was identified, the Sistrunk procedure was performed, which involves removal of the cyst along with the central portion of the hyoid bone to prevent recurrence [13]. Microscopic examination revealed a characteristic cyst lining composed of either squamous, pseudostratified ciliated columnar epithelium, or combination of both epitheliums (Table 1). It was often with surrounding thyroid follicles embedded in the stroma. The presence of thyroid follicles within the cyst wall was a key feature in distinguishing ITTDC from other cystic neck lesions, such as branchial cleft cysts or dermoid cysts in some cases. In the case reported by Chandraratnam *et al.*, mucinous gland was seen in the hypocellular fibrous cyst wall [2]. Follow-up data showed excellent outcomes, with no recurrences reported in patients who underwent complete excision. The follow-up period ranged from 6 months to 5 years, with most patients being symptom-free at their last clinical evaluation (Table 1). The incidence of malignancy in TGDCs is relatively rare, reported to be ⁓1%–1.5% of cases [1].There were no reported cases of ITTDC being associated with malignancy, which aligns with the general benign nature of TGDCs. However, a careful analysis of the thyrocyte nuclei is always necessary to avoid missing a papillary carcinoma developing within a TGDC, as malignant transformation, although very not reported yet, is still possible.

**Table 1:** Summary of reported cases in the literature.

**No.**	**Author**	**Pub. year**	**Country**	**Age (y)**	**Gender**	**Site**	**Clinical findings**	**US**	**Size (cm)**	**Biopsy & cytology**	**Surgery**	**Anatomical location subtype**	**Histology**	**Follow-up**
5	Hatada *et al.* [3]	2000	Japan	50	F	R	Lateral neck mass, discomfort on swallowing; cold on thyroid scan; low echoic mass on US	hypoéchoic nodule	4.4	US-FNAB: thick, viscous, grayish fluid; normal appearing squamous cells, no follicular cells	R lobectomy	I (completely surrounded by thyroid tissue, no evidence of TD remnants)	Squamous epithelium-lined cyst	Period NA, no recurrence
6	Johnston *et al.* [6]	2003	USA	10	M	L	Congenital anterior neck mass, palpable cyst in L thyroid, cold on thyroid scan, cystic on US	Cystic mass	3.5	‘Needle aspiration’: tan & mucoid material; CW a TDC	L hemithyroidectomy + isthmusectomy	I (completely embedded within the thyroid gland, no TD was noted)	FS: benign development remnant; FFPE: alternating respiratory & squamous epithelia-lined cyst, paucity of lymphoid tissue in the subepithelial region	18 months, no recurrence
8	Pérez-Martínez et al [11]	2005	Spain	11	M	R	Visible neck mass, cold on thyroid scan, cystic on US	Cystic mass	1.7	‘Biopsied’: obtained mucus and cells	Cyst excision	I (the superior half of R thyroid was replaced by the cyst; no adjacent fistulous tract or tributary was found)	Non-keratinized squamous epithelium and mono-stratified mucinous epithelium-lined cyst, islets of thyroid tissue in the wall	8 months, no recurrence
10	Álvarez Garcia *et al.* [12]	2015	Spain	2	M	R	Painless lateral neck mass, cold on scintigraphy, cystic on US	Cystic mass	1.7	Not done	‘The lesion was surgically resected’	I (in the thyroid)	Mucinous contenting cyst in the upper right lobe of the gland	NA
11	Álvarez Garcia *et al.* [12]	2015	Spain	10	M	R	Painless lateral neck mass; cold on scintigraphy, cystic on US	Cystic mass	2	FNAB: squamous epithelium, absence of colloid material or follicular tissue	Cyst excision	I (in the R thyroid)	Non-keratinized squamous epithelium-lined cyst, proteinaceous material inside	NA
12	Huang *et al.* [10]	2015	China	45	F	L	Bilateral neck mass along the midline; cystic on US	Cystic mass	4	Not done; instead, FS reported as a TDC	L hemithyroidectomy	I (separated nodule in L inferior pole of thyroid, no TD noted)	Pseudostratified ciliated columnar and squamous epithelia-lined cyst	NA
13	Saadi *et al.* [13]	2015	USA	48	M	Isthmus	Painless midline neck mass; cystic on US & CT	Cystic mass	1.1	FNAB: benign epithelial cells & macrophages	Cyst excision + Sistrunk	II (connected with a TD traced superiorly to the hyoid bone)	Epithelia-lined cyst with a thin, fibrous extension to the hyoid bone containing thyroid follicles	1 week, no recurrence
14	Barber *et al.* [9]	2018	USA	36	M	L	Acute thyroiditis with a tender neck mass; L neck mass on CT, thyroid complex cyst on US	thyroid complex cyst	5	FNAB: thick purulent material; acute inflammation, lymphohistiocytic tangles, bland appearing follicular cells; G+ cocci	L lobectomy	I (completely surrounded by thyroid tissue, with no external tract present)	Predominantly respiratory (ciliated, pseudostratified columnar) epithelium-lined cyst with focal squamous metaplasia and chronic inflammatory reaction	NA
18	Prabha *et al.* [4]	2020	India	25	F	L	Neck lump, gradually progressing in size; large thyroid cyst on US	Cystic mass	3.01	FNAB: dispersed benign squamous cells, few small sheets of follicular epithelial cells, numerous macrophages, colloid and neutrophils	L hemithyroidectomy	I (cystic lesion with surrounding thyroid tissue, no fistulous tract from the thyroid lobe)	Fibrocollagenous cyst wall lined by granulation tissue, hemosiderin laden macrophages and luminal anucleate squamous cells	9 months, no recurrence
19	Prabha *et al.* [4]	2020	India	41	M	L	Neck lump; large thyroid cyst on US	Cystic mass	4.9	FNAB: paucicellular smears with dispersed mature benign squamous cells and few anucleate squames on a clean background	L hemithyroidectomy	I (cystic ballooned out nodule in the left lobe of thyroid)	Fibrocollagenous cyst lined partly by cuboidal epithelium with predominantly denuded lining; thyroid follicles, thin blood vessels and chronic inflammation in the cyst wall	6 months, no recurrence
21	O’Neill *et al.* [5]	2021	USA	14	M	L	Neck swelling; a midline complex cystic structure near the L isthmus on US; a thick-walled, septated, complex fluid collection to the L of midline underneath the hyoid bone and extending inferiorly involving L lobe of thyroid near the junction of isthmus on CT	complex cyst	5	NA	L hemithyroidectomy + Sistrunk	II (cyst involving both L thyroid lobe and hyoid bone)	Cyst lined by pseudostratified ciliated columnar epithelium with focal squamous epithelium	6 months, no recurrence
22	Chandraratnam E et al [2]	NA	Australia	59	M	L	Hyperparathyroidism; incidentally identified a cystic lesion in L thyroid on US	cystic mass	1.5	US-FNAB: thick dark brown fluid; three epithelial fragments with no specific features in thick granular proteinaceous precipitate and abundant old blood	L hemithyroidectomy; L superior parathyroidectomy	I (completely within the left thyroid, no evidence of TD)	Cyst lined by pseudostratified, cuboidal to columnar and ciliated respiratory epithelium with one single small mucinous gland embedded in the hypocellular fibrocollagenous wall; L parathyroid gland: hypercellular parathyroid CW adenoma	2.5y, no recurrence
23	Present case	2024	Tunisia	47	F	L	Neck mass	Not detected	1.2	NA	L Hemithyroidectomy	I	1.2 cm cystic ductal structure was observed. It showed pseudostratified ciliated columnar and squamous epithelial lining associated with thyroid follicles in the surrounding stroma	6 months, no recurrence

## Conclusion

Intra-thyroid thyroglossal duct cysts (ITTDC) are very rare with only 23 documented cases in the literature. Pre-operative diagnosis is very challenging due to their unusual intra-thyroid location and nonspecific presentation. As highlighted by this case and the literature review, ITTDCs can be incidentally discovered or misdiagnosed as benign thyroid nodules, underscoring the importance of thorough histological examination. Imaging and fine-needle aspiration cytology often provide limited diagnostic clues, and the final diagnosis is made on microscopic examination. Despite their benign nature, careful examination of thyreocytes nuclei is warranted to exclude potential malignancy, although no cases of malignancy in ITTDC have been reported to date. Surgical excision remains the treatment of choice, with excellent outcomes and no recurrences documented in the reviewed cases.

## Data Availability

All data are available in the manuscript. Further enquiries can be directed to the corresponding author.
